# Optic, trigeminal, and facial neuropathy related to anti‐neurofascin 155 antibody

**DOI:** 10.1002/acn3.51220

**Published:** 2020-10-20

**Authors:** Hidenori Ogata, Xu Zhang, Saeko Inamizu, Ken‐ichiro Yamashita, Ryo Yamasaki, Takuya Matsushita, Noriko Isobe, Akio Hiwatashi, Shozo Tobimatsu, Jun‐ichi Kira

**Affiliations:** ^1^ Department of Neurology Neurological Institute Graduate School of Medical Sciences Kyushu University Fukuoka Japan; ^2^ Translational Neuroscience Center Graduate School of Medicine International University of Health and Welfare Okawa Japan; ^3^ Department of Clinical Neurophysiology Neurological Institute Graduate School of Medical Sciences Kyushu University Fukuoka Japan; ^4^ Department of Clinical Radiology Graduate School of Medical Sciences Kyushu University Fukuoka Japan; ^5^ School of Pharmacy at Fukuoka International University of Health and Welfare Okawa Japan; ^6^ Department of Neurology, Brain and Nerve Center Fukuoka Central Hospital International University of Health and Welfare Fukuoka Japan

## Abstract

**Objective:**

To characterize the frequency and patterns of optic, trigeminal, and facial nerve involvement by neuroimaging and electrophysiology in IgG4 anti‐neurofascin 155 antibody‐positive (NF155^+^) chronic inflammatory demyelinating polyneuropathy (CIDP).

**Methods:**

Thirteen IgG4 NF155^+^ CIDP patients with mean onset age of 34 years (11 men) were subjected to neurological examination, blink reflex, and visual‐evoked potential (VEP) testing, and axial and/or coronal T2‐weighted head magnetic resonance imaging (MRI).

**Results:**

Among 13 patients, facial sensory impairment, facial weakness, and apparent visual impairment were observed in three (23.1%), two (15.4%), and two (15.4%) patients, respectively. All 12 patients tested had blink reflex abnormalities: absent and/or delayed R1 in 11 (91.7%), and absent and/or delayed R2 in 10 (83.3%). R1 latencies had strong positive correlations with serum anti‐NF155 antibody levels (*r* = 0.9, *P* ≤ 0.0001 on both sides) and distal and F wave latencies of the median and ulnar nerves. Absent and/or prolonged VEPs were observed in 10/13 (76.9%) patients and 17/26 (65.4%) eyes. On MRI, hypertrophy, and high signal intensity of trigeminal nerves were detected in 9/13 (69.2%) and 10/13 (76.9%) patients, respectively, whereas optic nerves were normal in all patients. The intra‐orbital trigeminal nerve width on coronal sections showed a significant positive correlation with disease duration.

**Interpretation:**

Subclinical demyelination frequently occurs in the optic, trigeminal, and facial nerves in IgG4 NF155^+^ CIDP, suggesting that both central and peripheral myelin structures of the cranial nerves are involved in this condition, whereas nerve hypertrophy only develops in myelinated peripheral nerve fibers.

## Introduction

Chronic inflammatory demyelinating polyneuropathy (CIDP) is an acquired immune‐mediated disease of the peripheral nervous system (PNS) and constitutes the most prevalent chronic autoimmune neuropathy.^1^ For diagnosis of CIDP, electrodiagnostic evidence of primary demyelination is mandatory.^2^ Although CIDP usually affects the four limbs and results in sensorimotor disturbance in the extremities, the involvement of cranial nerves such as optic, oculomotor, trigeminal, and facial nerves has also been reported.^3^ Additionally, hypertrophy of cranial nerves in CIDP patients has occasionally been described in case reports or case series; however, its precise frequency has not been determined.^4^, ^5^, ^6^, ^7^, ^8^ Blink reflex testing, which measures the reflex time from stimulation of the afferent trigeminal supra‐orbital nerve to generation of a compound muscle action potential (CMAP) of the orbicularis oculi muscle by the efferent facial motor branch,^9^ is used to assess trigeminal and facial nerve function. Although blink reflex abnormalities in CIDP patients have been reported,^10^, ^11^, ^12^ their frequencies vary, which reflect CIDP’s etiological heterogeneity.^1^

Accumulating evidence indicates that some patients with CIDP harbor autoantibodies against nodal or paranodal proteins, such as neurofascin 186 (NF186), neurofascin 155 (NF155), contactin 1 (CNTN1), and contactin‐associated protein 1.^13^, ^14^, ^15^, ^16^ However, the frequency of either blink reflex abnormalities or trigeminal nerve hypertrophy has not been determined in anti‐NF155 antibody‐positive (NF155^+^) CIDP.

Although overt central nervous system (CNS) manifestations are rare in CIDP, CNS tissues including cerebral white matter and optic nerves could be involved. Various names have been used to describe such conditions, for example, combined central and peripheral demyelination (CCPD).^17^ We were the first to report the presence of anti‐NF155 antibodies in patients with CCPD.^18^ Subsequently, multiple sclerosis (MS)‐like white matter lesions in NF155^+^ CIDP were reported, whereas others did not detect anti‐NF155 antibodies in CCPD.^19^, ^20^ Thus, the CNS involvement in NF155^+^ CIDP is unclear and the differential effects of anti‐NF155 antibodies on peripheral myelin/Schwann cells and central myelin/oligodendrocytes remain to be established. Therefore, in this study, we aimed to characterize cranial nerve involvement in IgG4 NF155^+^ CIDP by neuroimaging and electrophysiology, with the trigeminal and facial nerves representing peripheral myelin structures and the optic nerve representing a central myelin structure.

## Subjects and Methods

### Subjects

Of 15 consecutive patients with IgG4 NF155^+^ CIDP diagnosed and treated in our department since 2001, 13 patients who underwent head magnetic resonance imaging (MRI) and blink reflex or visual‐evoked potential (VEP) testing were enrolled. All patients met the European Federation of Neurological Societies/Peripheral Nerve Society (EFNS/PNS) diagnostic criteria for CIDP.^2^ Distal acquired demyelinating symmetric neuropathy (DADS) was diagnosed according to Larue et al.^21^ The protocols for this study were approved by the Kyushu University Ethics Committee. All participants provided written informed consent.

### Anti‐nodal/paranodal autoantibody assay

IgG and IgG4 anti‐NF155 antibodies, and IgG anti‐NF186, and anti‐CNTN1 antibodies in sera, were measured by flow cytometry using human embryonic kidney 293 cell lines stably expressing human NF155, NF186, or CNTN1 as described previously.^14^, ^22^ In brief, equal numbers of transfected and untransfected cells were mixed and then incubated with patients’ sera. After washing, Alexa 647‐conjugated anti‐human IgG secondary antibodies were added. After incubation, cells were washed, resuspended in 100 µl phosphate‐buffered saline, and analyzed using a MACSQuant Analyzer (Miltenyi Biotec, Bergisch Gladbach, Germany). For each serum sample, the mean fluorescence intensity (MFI) ratio was calculated by dividing the Alexa 647 MFI of transfected cells by the Alexa 647 MFI of untransfected cells, whereas delta MFI was calculated by subtracting the Alexa 647 MFI of untransfected cells from the Alexa 647 MFI of transfected cells. Twelve sera taken around the time of blink reflex testing (within 1 month) were available for measuring anti‐NF155 antibody levels. Serum anti‐NF155 antibody levels are expressed using the MFI ratio and delta MFI, as previously described,^14^ to evaluate the correlation between ant‐NF155 antibody levels and blink reflex parameters. The time lag between blood withdrawal and blink reflex testing was 0.5 days (median, range 0–24 days). Sera at the time of brain MRI and VEP testing were not available in sufficient numbers for statistical analyses.

### Electrophysiological experiments

Nerve conduction studies (NCSs) were performed with standard surface stimulation and recording techniques (Nihon Kohden, Tokyo, Japan). Motor nerve conduction studies, including F wave analyses, were investigated in unilateral median and ulnar nerves. Skin temperature was maintained above 31°C in the upper limbs. Base‐to‐peak amplitudes were measured for CMAPs. Blink reflex was recorded with stimulation at the supra‐orbital notch and recording over the orbicularis oculi muscle on the inferior rim of the orbit directly below the pupil. Recordings were superimposed, and the shortest R1 and bilateral R2 latencies were used for each patient.^12^ Normal upper limits of R1, ipsilateral R2 (iR2) and contralateral R2 (cR2) for blink reflex were set as 13, 41, and 44 ms, respectively.^9^ Limb NCSs and blink reflex testing were performed within 2 weeks of each other. For VEPs, a checkerboard pattern was back‐projected onto a translucent screen. Stimulation was phase reversed at 1 Hz. The stimulating field subtended 8 degrees in diameter and the check sizes were 15 and 30 minutes of arc. The mean luminance was 180 cd/m^2^ with a contrast level of 90%. Subjects were instructed to fixate their eyes at the center of the stimulus field at a viewing distance of 100 cm. Monocular full visual fields were stimulated. VEPs were recorded from an electrode placed at Oz with a reference at Fz (International 10–20 system). A total of 100 responses were averaged and recordings were repeated at least twice to establish reproducibility. The latencies of the major positive peak in each check size were measured (P100 15’ and P100 30’). The upper normal limits (mean + 3 SD, ms) of VEPs were as follows; P100 15’, 123.8 ms; P100 30’, 121.0 ms.^23^

### Neuroimaging

Abnormalities of size and signal intensity in the first (V1) and second (V2) branches of trigeminal nerves in the orbit and the third branch (V3) at the level of the foramen ovale were evaluated by axial and/or coronal views of T2‐weighted images (T2WIs), including T2 spectral adiabatic inversion recovery images. Axial images were taken in all patients, whereas coronal images were taken in eight patients (Table 1). The cranial nerves of one patient (Case 8) were investigated by 3D nerve‐sheath signal increased with inked rest‐tissue rapid acquisition of relaxation enhancement imaging (3D SHINKEI). The median duration from the time of onset to head MRI was 63 months (range: 5 to 225 months). To investigate the relationship between nerve hypertrophy and disease duration, the maximum width of the intra‐orbital trigeminal nerve on each side on coronal MRI was measured. Cervical and lumbosacral nerve roots were investigated in all patients by magnetic resonance (MR) neurography. Abnormalities of optic and trigeminal nerves were judged by one neurologist (H.O.) and one radiologist (A.H.).

**Table 1:** Clinical and MRI findings in IgG4 NF155^+^ CIDP patients.

Case	1	2	3	4	5	6	7	8	9	10	11	12	13		
Sex	M	M	M	F	M	M	M	M	F	M	M	M	M	Sex ratio (M:F)	11:2
Age at onset (years)	16	67	13	58	47	17	34	26	16	48	50	31	25	Mean (years)	34
Clinical subtype	T	T	D	T	T	T	T	D	T	T	T	T	T	T: n/N (%)	11/13 (84.6)
Weakness of facial muscles	+	−	−	−	−	−	+	−	−	−	−	−	−	n/N (%)	2/13 (15.4)
Disturbance of facial sensation	−	+	−	−	−	−	+	+	−	−	−	−	−	n/N (%)	3/13 (23.1)
Decreased or absent corneal reflex	ND	−	ND	+	−	+	ND	−	ND	−	−	−	−	n/N (%)	2/9 (22.2)
CSF protein level (mg/dl)	412	217	205	255	369	334	328	454	282	227	103	320	327	Mean (mg/dl)	295
Hypertrophy of cervical nerve roots	+	+	+	+	+	+	+	+	+	+	+	+	+	n/N (%)	13/13 (100)
Hypertrophy of lumbosacral nerve roots	+	+	+	+	+	+	+	+	+	+	+	+	+	n/N (%)	13/13 (100)
Age at cranial MRI	17	68	14	60	50	20	39	32	26	58	64	48	41	Median (years)	41
Time from onset to head MRI (months)	5	6	9	27	31	37	63	73	117	126	159	210	225	Median (months)	63
Intra‐orbital hypertrophy of V1 and V2	−	−	−	−	+	−	−	+	+	−	+	+	+	n/N (%)	6/13 (46.2)
Intra‐orbital T2 hyperintensity of V1 and V2	−	−	−	−	+	−	−	+	+	−	+	+	+	n/N (%)	6/13 (46.2)
Hypertrophy of V3 at the level of the foramen ovale	−	−	+	+	−	−	+	+	+	−	−	−	+	n/N (%)	6/13 (46.2)
T2 hyperintensity of V3 at the level of the foramen ovale	−	−	+	+	+	−	+	+	+	+	−	+	+	n/N (%)	9/13 (69.2)
Optic nerve abnormality on coronal MRI	ND	−	ND	ND	−	−	ND	−	−	ND	−	−	−	n/N (%)	0/8 (0)
Brain lesions on MRI	−	+ (nonspecific)	−	−	+ (nonspecific)	+ (ovoid)	−	−	+ (ovoid)	−	−	−	^+^ (diffuse)	n/N (%)	3/13 (23.1)

CIDP, chronic inflammatory demyelinating polyneuropathy; D, distal acquired demyelinating symmetric neuropathy; CSF, cerebrospinal fluid; F, female; M, male; MRI, magnetic resonance imaging; n, number of positive patients; N, number of patients collated; NF155^+^, anti‐neurofascin 155 antibody positive; T, typical; V1, the first branch of the trigeminal nerve; V2, the second branch of the trigeminal nerve; V3, the third branch of the trigeminal nerve.

### Statistical Analysis

All analyses were performed in JMP Pro 15 (Cary, North Carolina, USA). Welch’s t‐test was used to compare the time from disease onset to MRI between patients with and without trigeminal nerve hypertrophy. The correlations between blink reflex and NCS results and other clinical parameters and between the maximum width of the intra‐orbital trigeminal nerve on each side and periods from disease onset to MRI were assessed using Pearson’s correlation coefficients.

## Results

### Anti‐nodal/paranodal antibodies and clinical features of participants

All 13 enrolled patients had serum IgG4 anti‐NF155 antibodies whereas none had serum anti‐NF186 or anti‐CNTN1 antibodies. The mean age at onset was 34 years of age and 11 patients were male **(**Table 1). Eleven had typical CIDP, whereas two were classified as DADS.^21^ Two patients (Cases 4 and 10) had a medical history of glaucoma and cataract, whereas another (Case 1) had an episode of transient blurred vision at 5 months from onset. Two patients (Cases 4 and 13; 15.4%) complained of mild uncorrectable visual impairment at the time of VEP testing. Impaired facial sensation and facial weakness at the time of blink reflex testing were found in three (Cases 2, 7, and 8; 23.1%) and two (Cases 1 and 7; 15.4%) patients, respectively. Corneal reflex was diminished in two (Cases 4, and 6) of nine patients evaluated. The mean CSF protein level was 295 mg/dl. All 13 patients showed hypertrophy of cervical and lumbosacral nerve roots on MR neurography. On head MRI, two young patients (Cases 6 and 9; 15.4%) had T2‐hyperintense ovoid lesions suggestive of demyelination, two middle‐ and old‐aged patients (Cases 2 and 5) had multiple nonspecific T2‐hyperintense deep white matter lesions, and another young patient (Case 13) showed diffuse symmetric T2‐hyperintense white matter lesions at the acute phase.

### Abnormalities in the R1 and R2 responses of the blink reflex

**Figure 1:**
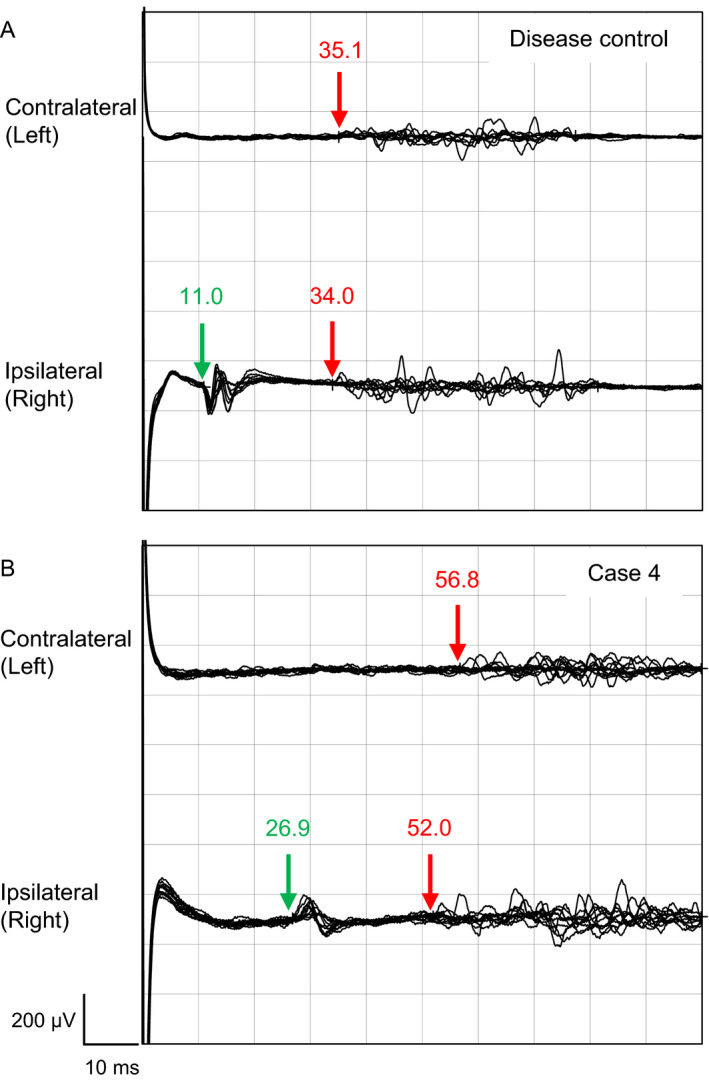
Representative blink reflex traces in a patient with IgG4 NF155^+^ CIDP. Compared with a disease control 63‐year‐old woman with multifocal acquired demyelinating sensory and motor neuropathy (A), the latencies of R1 and ipsilateral and contralateral R2 are clearly prolonged in Case 4 with IgG4 NF155^+^ CIDP (B).

R1 abnormalities were observed in all but one of 12 patients examined (Case 10; 91.3%) and in 21 of 24 sides (87.5%) tested (Fig. 1 and Table 2). Bilateral prolonged R1 latencies were observed in eight patients (Cases 3, 4, 5, 7, 9, 11, 12, and 13), whereas R1 responses were not elicited bilaterally in Case 8. Case 2 had prolonged R1 latency on the right side and no evoked potential on the other side. Case 6 showed the prolonged R1 latency on the left side and just the normal upper limit latency on the right side. Abnormal (absent and/or delayed) iR2 and cR2 were seen in 19 (79.2%) and 17 (70.8%) of 24 tests, respectively (Table 2). Case 10 with normal R1 latencies presented with abnormal R2 responses. Overall, all but one patient (Case 6) had abnormal findings on R2 responses. Therefore, all 12 patients tested had abnormalities in the R1 and/or R2 responses of the blink reflex.

**Table 2:** Blink reflex findings in IgG4 NF155^+^ CIDP patients.

	Case	1	2	3	4	5	6	7	8	9	10	11	12	13	Median	Abnormal/N (%)	Median	Abnormal/N (%)
	Age at blink reflex test (years)	ND	67	19	60	50	20	40	31	26	60	64	48	43	45.5			
	Time from onset to blink reflex test (months)	ND	5	72	24	31	37	69	62	117	147	158	210	217	70.5			
R1 (ms)	Left	ND	**NE**	**17.3**	**27.5**	**30.6**	**13.5**	**30**	**NE**	**25.7**	11.9	**14.2**	**22.2**	**15.8**	19.8	11/12 (91.7)	19.8	21/24 (87.5)
	Right	ND	**19.8**	**16.8**	**26.9**	**31.3**	12.9	**30.9**	**NE**	**23.4**	12.2	**13.4**	**19.9**	**17.4**	19.8	10/12 (83.3)		
iR2 (ms)	Left	ND	**51.4**	**61.6**	**57.6**	**57.2**	37.8	**47.8**	**NE**	**51.5**	**43.2**	**42.8**	**59.8**	36.7	51.4	10/12 (83.3)	47.8	19/24 (79.2)
	Right	ND	**44.7**	**NE**	**52**	**62.7**	38.3	**56.9**	**NE**	**53.6**	**44**	35.3	**46**	39.8	45.4	9/12 (75.0)		
cR2 (ms)	Left	ND	**45.7**	**63.6**	**56.8**	**54.4**	40.6	**48.9**	**NE**	**NE**	**NE**	40.5	**51**	40.2	48.9	9/12 (75.0)	48.9	17/24 (70.8)
	Right	ND	**53.2**	**NE**	**56.8**	**NE**	37.4	**58.1**	**NE**	**NE**	43.6	33.8	**52.6**	39.7	48.1	8/12 (66.7)		

Normal upper limits of R1, iR2, and cR2 in the blink reflex test were set as 13, 41, and 44 ms, respectively.^9^ Abnormal values are indicated in bold. Case 8, whose R1 responses were not elicited bilaterally, had shown bilateral prolonged R1 latencies (right: 26.1 ms, left: 26.9 ms) in a prior test at a different institution four months after onset.

CIDP, chronic inflammatory demyelinating polyneuropathy; cR2, contralateral R2; iR2, ipsilateral R2; N, number of examinations; ND, not done; NE, not evoked; NF155^+^, anti‐neurofascin 155 antibody positive.

### Abnormalities of VEP P100

**Figure 2:**
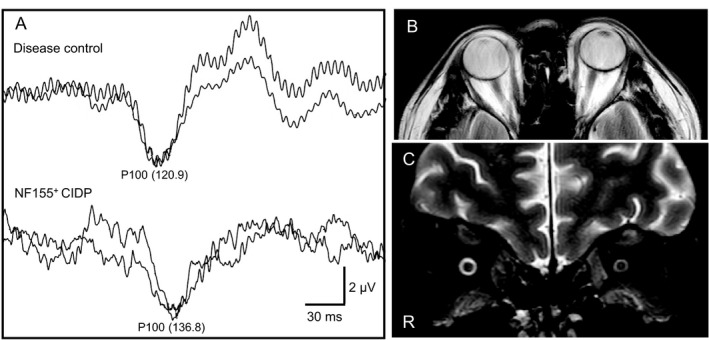
Representative traces of visual‐evoked potentials in a patient with IgG4 NF155^+^ CIDP. Compared with a disease control 40‐year‐old man with atopic myelitis (120.9 ms, upper raw in A), the right eye of Case 5 with IgG4 NF155^+^ CIDP shows delayed P100 15’ latency (136.8 ms, lower raw in A). Case 5 does not show any optic nerve abnormalities on MRI (B and C).

Analysis of P100 15’ showed 10/13 patients (76.9%) and 17/26 eyes (65.4%) to have abnormalities (Fig. 2A and Table 3). Prolongation of unilateral and bilateral P100 15’ was seen in three (Cases 4, 6, and 9) and four (Cases 2, 5, 10, and 11) patients, respectively. No potentials were evoked in two patients (Cases 8 and 12). Another patient (Case 13) showed absent and prolonged P100 15’ in each eye. Analysis of P100 30’ showed 7/13 patients (53.8%) and 13/26 eyes (50.0%) to have absent or prolonged evoked potentials (Table 3). Prolongation of unilateral and bilateral P100 15’ was seen in one (Case 5) and four patients (Cases 2, 6, 10, and 13), respectively. No evoked potentials were detected in one patient (Case 8). Another patient (Case 12) showed absent and prolonged P100 30’ in different eyes. Overall, VEPs were prolonged or unevoked in 10/13 (76.9%) patients and in 17/26 (65.4%) eyes.

**Table 3:** VEP findings in IgG4 NF155^+^ CIDP patients.

Case		1	2	3	4	5	6	7	8	9	10	11	12	13	Median	Abnormal/N (%)	Median	Abnormal/N (%)
Age at VEP test (years)		19	68	14	61	50	20	35	32	26	59	59	48	25	35			
Visual acuity at VEP test^1^		0	0	0	−1	0	0	0	0	0	0	0	0	−1	0			
Time from onset to VEP test (months)		33	5	9	33	31	37	10	71	117	147	104	211	3	33			
P100 15’ (ms)	Left eye	108.3	**139.2**	111.3	123.3	**133.2**	122.1	120.6	**NE**	**124.8**	**151.5**	**132.6**	**NE**	**150**	124.8	8/13 (61.5)	125.1	17/26 (65.4)
	Right eye	111.9	**137.1**	109.2	**125.1**	**136.8**	**132.9**	117.9	**NE**	114.6	**150.3**	**139.8**	**NE**	**NE**	129	9/13 (69.2)		
P100 30’ (ms)	Left eye	106.5	**125.7**	102.3	108	114.3	**121.5**	116.1	**NE**	103.8	**142.5**	119.1	**NE**	**163.5**	116.1	6/13 (46.2)	119.1	13/26 (50.0)
	Right eye	105.9	**125.1**	107.4	110.4	**124.2**	**125.1**	112.8	**NE**	106.2	**154.5**	120	**126.3**	**156**	122.1	7/13 (53.8)		

Abnormal values are indicated in bold. CIDP, chronic inflammatory demyelinating polyneuropathy; N, number of eyes examined; NE, not evoked; ND, not done; NF155^+^, anti‐neurofascin 155 antibody‐positive; VEP, visual‐evoked potential.

^1^ Visual acuity was scored as follows: 0, normal; −1, mild vision impairment; −2, finger counting; −3, light perception; −4, total blindness.

### Hypertrophy and signal abnormality of the trigeminal nerves on MRI

**Figure 3:**
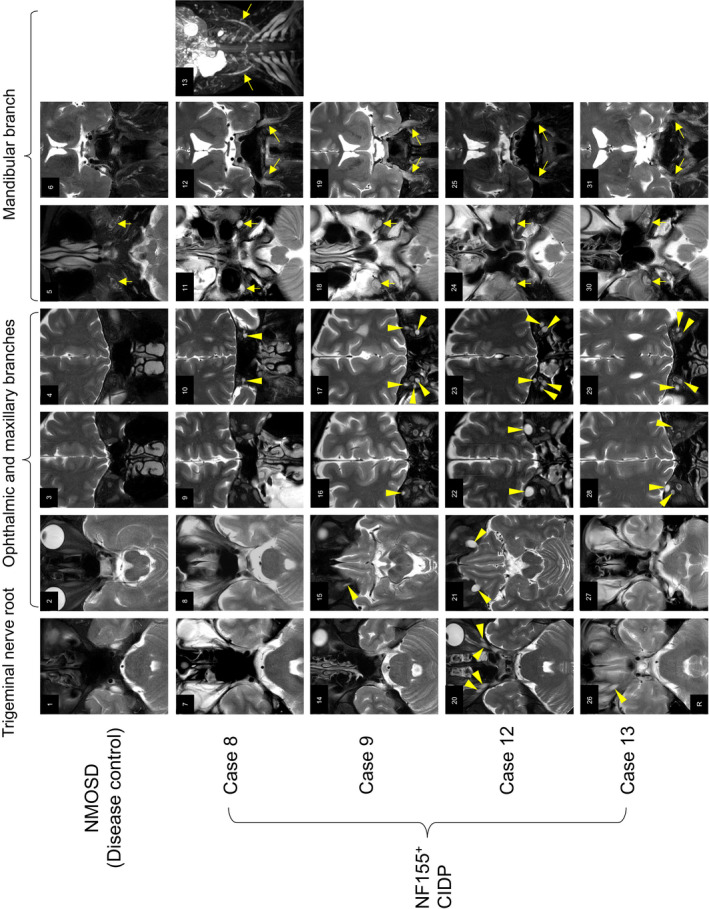
Hypertrophy and signal abnormality of trigeminal nerves in patients with IgG4 NF155^+^ CIDP. Head MRI of a 53‐year‐old female disease control patient with neuromyelitis optica spectrum disorder (NMOSD) (**1–6**) and of NF155^+^ CIDP patients (Case 8, **7–13**; Case 9, **14–19**; Case 12, **20–25**, and Case 13, **26–31**) are shown. Trigeminal nerve roots (the emitting portion of the trigeminal nerve from the brainstem) (**1**, **7**, **14**, **20**, **26**), intra‐orbital ophthalmic and maxillary branches (**2–4, 8–10, 15–17, 21–23,** and **27–29**) and mandibular branches at the level of the foramen ovale are shown in axial (**5**, **11**, **18, 24 and 30**) and coronal (**6, 12, 19, 25,** and **31**) views. The ophthalmic and maxillary branches (arrowheads) and mandibular branch (arrows) are identified in all NF155^+^ CIDP cases whereas these branches are hardly visible in the control. The mandibular branches (arrows) at the level of the foramen ovale have higher signal intensity and/or hypertrophy in Cases 8 (**11** and **12**), 9 (**18** and **19**), 12 (**24** and **25**), and 13 (**30** and **31**) compared with the control (**5** and **6**) (arrows). The coronal view of 3D SHINKEI also demonstrates hypertrophic mandibular branches in Case 8 (**13**). Abbreviations: CIDP = chronic inflammatory demyelinating polyneuropathy, NF 155^+^ = anti‐neurofascin 155 antibody positive, Rt = right.

Among 13 patients examined, 10 (76.9%) had abnormal findings of the trigeminal nerves on axial and/or coronal T2WIs (Fig. 3 and Table 1). Six patients (46.2%) showed intra‐orbital hypertrophy of the ophthalmic and maxillary branches. These hypertrophic nerves commonly exhibited hyperintensity on T2WIs (Fig. 3). One patient (Case 12) had diplopia because of compression of the left superior rectus muscle caused by a massively enlarged supra‐orbital nerve (Fig. 3). Additionally, the mandibular branch at the level of the foramen ovale showed high signal intensity on T2WIs in nine patients (69.2%), whereas its hypertrophy was evident in six patients (46.2%; Fig. 3). Case 8, evaluated by 3D SHINKEI, demonstrated hypertrophy of the mandibular branch (Fig. 3). Overall, hypertrophy and high signal intensity in the three branches of the trigeminal nerve were observed in 9/13 (69.2%) and 10/13 (76.9%) patients, respectively, whereas the emitting portion of the trigeminal nerve from the brainstem never showed hypertrophy (Fig. 3).

### Optic nerves on MRI

No morphological or signal abnormalities were found in the optic nerves of any of the 13 patients, including the eight patients with VEP abnormalities who were also evaluated by coronal T2WI (Fig. 2B, C and Table 1).

### Correlation between blink reflex abnormalities and other clinicolaboratory parameters

Among the 12 patients subjected to the blink reflex test, all three patients with trigeminal sensory impairment, including one with additional facial weakness, had blink reflex abnormalities. Moreover, all nine patients with trigeminal nerve hypertrophy had blink reflex abnormalities. Nine of the twelve patients had both blink reflex and VEP abnormalities, whereas two patients (Cases 3 and 7) had an abnormal blink reflex but normal VEPs and one patient (Case 10) had a normal blink reflex but abnormal VEPs. Parameters of the blink reflex did not correlate with age at onset, age at blink reflex test or the time from disease onset to the blink reflex test.

**Figure 4:**
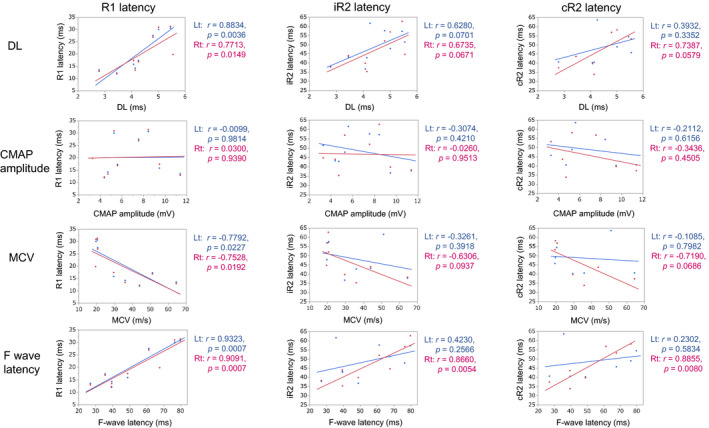
Correlation between blink reflex results and ulnar NCS parameters in IgG4 NF155^+^ CIDP. Correlations between R1, iR2, and cR2 latencies and NCS parameters (DL, CMAP, MCV, and F wave latency) are shown. CIDP, chronic inflammatory demyelinating polyneuropathy; CMAP, compound muscle action potential; DL, distal latency; Lt, left; MCV, motor conduction velocity; NCS, nerve conduction study; NF 155^+^, anti‐neurofascin 155 antibody‐positive; Rt, right.

**Figure 5:**
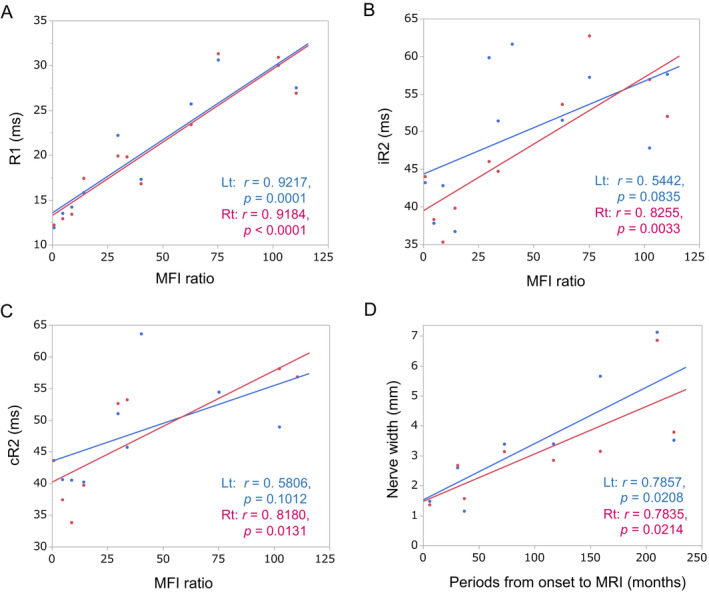
Correlations between blink reflex parameters and anti‐NF155 antibody levels and between trigeminal nerve hypertrophy and disease duration in IgG4 NF155^+^ CIDP. Correlations of anti‐NF155 antibody levels with R1, iR2, and cR2 latencies on stimulation of either side are shown in A, B, and C, respectively. Anti‐NF155 antibody levels are expressed as MFI ratios.^14^ (D) shows a significant positive correlation between the maximum width of the intra‐orbital trigeminal nerve on each side and the period from onset to MRI. CIDP, chronic inflammatory demyelinating polyneuropathy; HC, healthy controls; Lt, left; MFI, mean fluorescence intensity; NF 155^+^, anti‐neurofascin 155 antibody positive; Rt = right.

Correlation analysis of the blink reflex and ulnar nerve NCS parameters showed that bilateral R1 latencies positively correlated with distal latencies (DLs; right: *r* = 0.7713, *P* = 0.0149; left: *r* = 0.8834, *P* = 0.0036) and F wave latencies (right: *r* = 0.9091, *P* = 0.0007; left: *r* = 0.9323, *P* = 0.0007), and negatively correlated with motor conduction velocities (MCVs; right: *r* = −0.7528, *P* = 0.0192; left; *r* = −0.7792, *P* = 0.0227; Fig. 4). However, there was no correlation between R1 blink reflex parameters and CMAP amplitudes. For R2 parameters with right side stimulation, both iR2 and cR2 latencies positively correlated with F wave latencies (iR2: *r* = 0.8660, *P* = 0.0054; cR2: *r* = 0.8855, *P* = 0.0080), and tended to positively correlate with DLs (iR2: *r* = 0.6735, *P* = 0.0671; cR2: *r* = 0.7387, *P* = 0.0579) and to negatively correlate with MCVs (iR2: *r* = −0.6306, *P* = 0.0937; cR2: *r* = −0.7190, *P* = 0.0686). Neither iR2 nor cR2 latencies showed correlation with CMAP amplitudes. Similar trends were observed with left side stimulation, albeit they were not significant. Similar correlations were also found between the blink reflex and median nerve NCS parameters (data not shown). Correlation analysis of the blink reflex parameters and serum anti‐NF155 antibody levels revealed that R1 latencies on stimulation of either side (right: *r* = 0.9184, *P* < 0.0001; left; *r* = 0.9217, *P* = 0.0001) and iR2 and cR2 latencies on right side stimulation (iR2: *r* = 0.8255, *P* = 0.0033; cR2: *r* = 0.8180, *P* = 0.0131) were positively correlated with MFI ratios (Fig. 5A–C) and delta MFI (data not shown).

### Correlation between VEP abnormalities and other clinicolaboratory parameters

When the relationship between VEPs and other clinicolaboratory parameters was investigated, P100 15’ latencies of the right eye positively correlated with age at onset and age at VEP examination (age at onset: *r* = 0.6832, *P* = 0.0294; age at VEP: *r* = 0.7462, *P* = 0.0132). However, no correlation between VEP abnormalities and somatic NCS parameters was found.

### Correlation between trigeminal nerve hypertrophy and other clinicolaboratory parameters

Disease duration was more than two‐fold longer in patients with trigeminal nerve hypertrophy than in those without, although the difference did not reach statistical significance (102 ± 81 months vs. 44 ± 57 months, *P* = 0.1752). Additionally, the intra‐orbital trigeminal nerve width on coronal sections showed a significant positive correlation with disease duration (right, *r* = 0.7835, *P* = 0.0214; left side, *r* = 0.7857, *P* = 0.0208; Fig. 5). There was no correlation between trigeminal nerve hypertrophy and other clinicolaboratory parameters.

## Discussion

The main findings of this study are as follows. First, a high frequency of patients with NF155^+^ CIDP had subclinical blink reflex and VEP abnormalities, suggestive of demyelination of the optic, trigeminal, and facial nerves. Second, nerve hypertrophy and high signal intensity were frequently observed in the trigeminal nerves but almost no abnormalities were detectable in the optic nerves by MRI in patients with NF155^+^ CIDP. Third, blink reflex parameters, particularly R1 latencies, were strongly positively correlated with distal and F wave latencies of the somatic nerves as well as serum anti‐NF155 antibody levels.

Concerning cranial nerve involvement in CIDP, blink reflex testing has repeatedly been shown to be helpful. Blink reflex test abnormalities in CIDP were reported to occur in 53.3% (8/15)^10^ and 62.1% (36/58)^12^ of Caucasians and in 90% (18/20) of Japanese.^11^ In these previous studies, anti‐NF155 antibodies were not examined. However, the prevalence of anti‐NF155 antibodies is reported to be lower in Western countries (1% to 10% positive)^24^, ^25^, ^26^ compared with Asian countries (18% and 21% positive);^14^, ^27^ therefore, it is conceivable that the results of these previous reports mostly reflected anti‐NF155 antibody‐negative (NF155^‐^) CIDP cases, particularly in the reports of Caucasians. Given that all NF155^+^ CIDP patients examined in this study had abnormal blink reflex, a higher frequency of blink reflex abnormalities is indicated for NF155^+^ compared with NF155^‐^ CIDP. The relatively higher frequency of blink reflex abnormalities in Japanese total CIDP patients^11^ compared with Caucasian total CIDP patients^10^, ^12^ may partly reflect a higher frequency of NF155^+^ CIDP in Asians^14^, ^27^ compared with Caucasians.^24^, ^25^, ^26^ Thus, the higher frequency of prolonged R1 and R2 latencies in blink reflex testing without overt facial or trigeminal manifestations indicates that subclinical demyelination of these nerves is a common feature of NF155^+^ CIDP.

Interestingly, our study showed a significant positive correlation of serum anti‐NF155 antibody levels with various blink reflex parameters, which suggests that high titer serum anti‐NF155 antibodies worsen conduction delay in the trigeminal and facial nerves more severely than low titer anti‐NF155 antibodies. This is consistent with the previous observation that anti‐NF155 antibody levels changed in parallel with MCV and F wave latencies in somatic nerves.^28^ Therefore, these findings collectively support the pathogenic role of anti‐NF155 antibodies in this condition.

A much higher frequency of R1 abnormality in the blink reflex test than trigeminal nerve hypertrophy indicates that subclinical demyelination precedes nerve hypertrophy.

The high frequency of VEP abnormalities is rather surprising, given that most NF155^+^ CIDP patients do not complain of visual disturbance, as described here and in previous reports.^14^ VEP abnormalities were found in 47% (8/17)^29^, 50% (9/18)^30^, and 50% (5/10)^31^ of total Caucasian CIDP patients. Thus, the abnormal VEP frequency of 76.9% in the present NF155^+^ CIDP series is higher than the reported abnormal VEP frequencies in total CIDP patients,^29^, ^30^, ^31^ which mostly reflects NF155^‐^ CIDP cases, given the low frequency of NF155^+^ CIDP in Caucasians.^24^, ^25^, ^26^

For VEP analysis, the monocular full visual field was stimulated; therefore, marked prolongation of P100 latencies in the absence of ophthalmological retinal lesions and occipital MRI lesions suggests the presence of subclinical demyelination in the optic nerves in most of our cases. NF155 is expressed in the terminal loop of not only Schwan cells but also oligodendrocytes;^32^ therefore, it is reasonable that the optic nerves can be frequently affected by anti‐NF155 antibodies. The present findings are consistent with previous reports describing the occurrence of CNS white matter lesions suggestive of demyelination in a fraction of NF155^+^ CCPD and CIDP patients.^18^, ^20^ The high prevalence of optic, trigeminal, and facial nerve demyelination is a unique feature of NF155^+^ CIDP, which could be overlooked in clinical practice unless the blink reflex and VEPs are examined.

The analysis of correlation between cranial and somatic NCS findings revealed that blink reflex parameters, particularly R1 latencies, were strongly correlated with NCS findings in the ulnar and median nerves, whereas no correlation existed between VEP and NCS findings. We therefore suggest that both the PNS and CNS are commonly afflicted in NF155^+^ CIDP, but that PNS and CNS involvement does not proceed in parallel. The observation that the correlation between R1 latency and NCS parameters seems to be stronger than that between R2 latencies and NCS parameters may be explained by longer CNS portions in the R2 compared with the R1 response.^9^ Normal R1 responses but prolonged R2 responses in Case 10 may reflect subclinical involvement of the brainstem. Among somatic NCS parameters, DL‐ and F‐wave latencies correlated with blink reflex abnormalities more strongly than MCV and CMAP amplitudes. In NF155^+^ CIDP, DL‐ and F‐wave latencies are affected most severely,^14^ probably because anti‐NF155 antibodies enter PNS tissue at nerve terminals and spinal roots where the blood–nerve barrier is anatomically absent or loose. Thus, similar antibody‐mediated nerve terminal damage is likely to occur in the trigeminal and facial nerves in NF155^+^ CIDP.

Our study clearly shows that in NF155^+^ CIDP, nerve hypertrophy frequently occurs in the trigeminal nerve branches but not in the optic nerves even in the same intra‐orbital cavity, although both nerves suffer from subclinical demyelination with similarly high frequency. The involvement of cranial nerve hypertrophy only in the PNS portion is also supported by our observation that the initial segments of the trigeminal nerve covered by oligodendrocytes did not show hypertrophy in any patient. Cranial nerve hypertrophy is sometimes visible on MRI in CIDP.^4^, ^5^, ^6^, ^7^, ^8^ Following our report describing high frequency of prominent hypertrophy of cervical and lumbosacral nerve roots and proximal segments of peripheral nerves in NF155^+^ CIDP,^14^ hypertrophy of oculomotor and trigeminal nerves was reported in a NF155^+^ CIDP case.^33^ The results of this study indicating trigeminal nerve hypertrophy involving all three branches are the common feature of NF155^+^ CIDP with a longstanding clinical course, which is helpful for suspecting this condition. Hypertrophy of cervical and/or lumbosacral nerve roots was seen in all 13 patients examined, whereas trigeminal nerve hypertrophy was detectable in around three‐quarters of the patients, which indicates that somatic nerve hypertrophy precedes cranial nerve hypertrophy. As in the somatic nerve,^14^ trigeminal nerve hypertrophy was associated with longer disease duration. Thus, nerve hypertrophy in NF155^+^ CIDP is suggested to gradually proceed over a long disease course in the cranial nerves belonging to the PNS but not the CNS, along with somatic nerve hypertrophy.

Pathology of hypertrophic intra‐orbital nerves from some CIDP patients demonstrated onion bulb formation reflecting repeated demyelination and remyelination, infiltration of inflammatory cells, and abundant mucopolysaccharide deposition.^4^, ^34^ Although sural nerve biopsied from NF155^+^ CIDP patients showed only perineurial and endoneurial edema without onion bulb formation or inflammatory cell infiltration,^14^, ^35^, ^36^ nothing is known about the pathology of the hypertrophic parts of somatic or cranial nerves. Anti‐NF155 antibodies are assumed to invade the distal nerve terminals and nerve roots where the blood–nerve barrier is absent or loose, and to induce axo‐glial detachment at the node of Ranvier.^35^ However, it is unclear how such axo‐glial detachment causes nerve hypertrophy in this condition. We recently reported marked increases in the levels of proinflammatory cytokines in cerebrospinal fluid (CSF) from patients with NF155^+^ CIDP,^37^ which may cause blood–nerve barrier disruption at the nerve roots resulting in marked increases in CSF protein levels. Thus, prominent nerve edema, as seen in the biopsied sural nerve,^14^ which can be triggered by upregulated proinflammatory cytokines, may be responsible in part for somatic and cranial nerve hypertrophy in this disease. However, it is difficult to explain the lack of nerve hypertrophy in the CNS portion of the cranial nerves solely by inflammation‐related edema. Further pathological and experimental studies are required to elucidate the mechanism of nerve hypertrophy involving only PNS but not CNS tissues.

This study has several limitations. First, this is a retrospective study; therefore, slice thickness was not unified for the evaluation of cranial nerve hypertrophy and some clinicolaboratory data, such as corneal reflex and coronal T2WIs, were not available in several patients. Thus, clinical manifestations and nerve hypertrophy of cranial nerves could be under‐estimated. Second, the sample number was small because of the rarity of IgG4 NF155^+^ CIDP. Thus, our study results should be confirmed by future large‐scale studies. Third, as we could not obtain sufficient data on blink reflex, VEP or brain MRI in NF155^‐^ CIDP patients, we compared between historical study results of total CIDP patients and NF155^+^ CIDP data. Thus, abnormality frequencies in these tests should be compared between NF155^+^ and NF155^‐^ CIDP patients in future research. Fourth, we did not have sufficient longitudinal blink reflex or VEP data for NF155^+^ CIDP. Thus, changes in subclinical demyelination in the optic, trigeminal, and facial nerves in response to immunotherapy should be longitudinally examined in the future.

Nonetheless, we uncovered high‐frequency subclinical blink reflex and VEP abnormalities as well as trigeminal nerve hypertrophy, which suggest homogeneity of NF155^+^ CIDP. Our study underscores the importance of awareness of the frequent involvement of the cranial nerves, including optic, trigeminal, and facial nerves, in NF155^+^ CIDP to prevent long‐term damage to these cranial nerves by immunotherapy.

## Conflicts of interest

Nothing to report.

## Authors’ contributions

HO, XZ, SI, KY, RY, TM, NI, and JK designed and conceptualized the study, collected the data, analyzed the data, and drafted the manuscript. AH and ST analyzed the data and drafted the manuscript.
